# Artificial intelligence and hybrid imaging: the best match for personalized medicine in oncology

**DOI:** 10.1186/s41824-020-00094-8

**Published:** 2020-12-09

**Authors:** Martina Sollini, Francesco Bartoli, Andrea Marciano, Roberta Zanca, Riemer H. J. A. Slart, Paola A. Erba

**Affiliations:** 1 Department of Biomedical Sciences, Humanitas University, Pieve Emanuele (Milan), Italy; 2 Humanitas Clinical and Research Center, Rozzano (Milan), Italy; 3 Regional Center of Nuclear Medicine, Department of Translational Research and New Technologies in Medicine and Surgery, University of Pisa, Pisa, Italy; 4 University Medical Center Groningen, Medical Imaging Center, University of Groningen, Groningen, The Netherlands; 5 Faculty of Science and Technology, Biomedical Photonic Imaging, University of Twente, Enschede, The Netherlands

**Keywords:** Artificial intelligence, Hybrid imaging, Deep learning, Machine learning, Natural language processing, Distributed learning, Radiomics, Computer-aided diagnosis systems, PET/CT, Imaging biomarkers

## Abstract

Artificial intelligence (AI) refers to a field of computer science aimed to perform tasks typically requiring human intelligence. Currently, AI is recognized in the broader technology radar within the five key technologies which emerge for their wide-ranging applications and impact in communities, companies, business, and value chain framework alike. However, AI in medical imaging is at an early phase of development, and there are still hurdles to take related to reliability, user confidence, and adoption. The present narrative review aimed to provide an overview on AI-based approaches (distributed learning, statistical learning, computer-aided diagnosis and detection systems, fully automated image analysis tool, natural language processing) in oncological hybrid medical imaging with respect to clinical tasks (detection, contouring and segmentation, prediction of histology and tumor stage, prediction of mutational status and molecular therapies targets, prediction of treatment response, and outcome). Particularly, AI-based approaches have been briefly described according to their purpose and, finally lung cancer—being one of the most extensively malignancy studied by hybrid medical imaging—has been used as illustrative scenario. Finally, we discussed clinical challenges and open issues including ethics, validation strategies, effective data-sharing methods, regulatory hurdles, educational resources, and strategy to facilitate the interaction among different stakeholders. Some of the major changes in medical imaging will come from the application of AI to workflow and protocols, eventually resulting in improved patient management and quality of life. Overall, several time-consuming tasks could be automatized. Machine learning algorithms and neural networks will permit sophisticated analysis resulting not only in major improvements in disease characterization through imaging, but also in the integration of multiple-omics data (i.e., derived from pathology, genomic, proteomics, and demographics) for multi-dimensional disease featuring. Nevertheless, to accelerate the transition of the theory to practice a sustainable development plan considering the multi-dimensional interactions between professionals, technology, industry, markets, policy, culture, and civil society directed by a mindset which will allow talents to thrive is necessary.

## Background

**Fig. 1:**
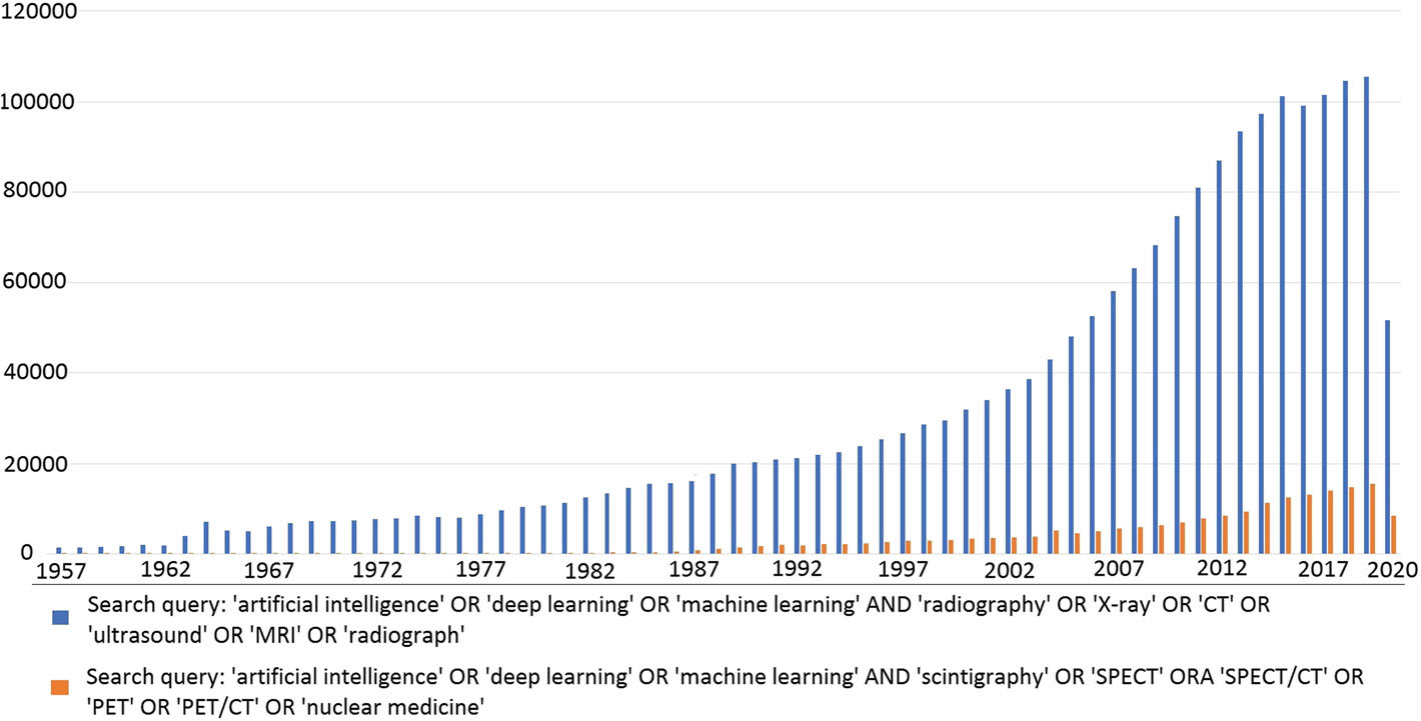
Temporal trend of publications on AI-based applications in radiology and nuclear medicine imaging

Artificial intelligence (AI) refers to a field of computer science aimed to perform tasks typically requiring human intelligence (Chartrand et al. 2017). The first publication on AI (early neural network) was on chess, and dates back to 1943. In 1957, AI was founded as an academic discipline, and thereafter it underwent an evolution until its most modern concept. However, after a doubtless period of enthusiasm and growth, AI experienced a “winter” of reduced funding and interest (1970s–1990s), mainly due to a stall in progress as result of disappointments in machine translation, and the fall of the connectionist movement as consequence of the belief that there was no algorithm to train a network with multiple layers able to solve the non-linear separable problems. In the late 1990s and in the early 2000s, non-neural machine learning methods (e.g., support vector machine) dominated many commercial pattern recognition applications. At the same time, it has been demonstrated that, at least in some domains, neural networks outperformed other techniques (Schmidhuber 2015; Minsky and Papert 1972). Overall, these events together with new technologies (e.g., Fast Graphics Processing Units), the development of more sophisticated and efficient AI systems, and the digital transformation of data in “big data” resulted in a renew interest in the discipline, as shown by investments, funding, and research being in the following years (Schmidhuber 2015; Sollini et al. 2019a). Currently, AI is recognized in the broader technology radar within the five key technologies which emerge for their wide-ranging applications and impact in communities, companies, business, and value chain framework alike. As AI, medicine has undergone over the years the same adventure, being increasingly “technological” (e.g., robotic surgery, hybrid imaging), “efficient” (i.e., personalized medicine), and “digital” (e.g., electronic medical report, PACS). Imaging, certainly, has been one of the areas of greatest development in this regard. Therefore, the growing interest in AI applications to medical imaging should not be surprising. However, it should be acknowledged that AI-based applications in radiology preceded nuclear medicine imaging (Fig. 1). Some reasons are possibly related to the premature coming of AI in radiology with respect to nuclear medicine. Firstly, AI approaches typically need of large dataset, and numbers in radiology (generally more accessible, faster, and sometimes easier) have been always higher than in nuclear medicine. Secondly, nuclear medicine has been, at least in the past, doubtless more variable and less standardized than radiology (different tracers, acquisition protocols, types of images/views according to availability, clinical question, and patient’s characteristics), resulting in a lower intra- and inter-method consistency. Nonetheless, technology, standardization, and availability have great leap forward in the past years, making nuclear medicine one of the most attractive fields of application of AI approaches.

The present narrative review aimed to provide an overview on AI-based approaches in oncological hybrid medical imaging with respect to purposes and clinical settings. Particularly, AI-based approaches have been briefly described according to their purpose and, finally lung cancer—being one of the most extensively malignancy studied by hybrid medical imaging—has been used as illustrative scenario.

### AI-based approaches: purposes

AI has stand out as a compelling tool for integrated analysis of the rapidly developing of multi-omics data, including many research and clinical tasks such as prediction of disease aggressiveness and identification of mutational status and molecular therapies targets (Moore et al. 2019). In medical imaging, AI can be used with different purposes from secure system for data-sharing (distributed learning), to statistical analysis (statistical learning), to computer-aided diagnosis systems, and other fully automated image analysis tool able to integrate other data supporting clinical decision (Clinical Decision Support Systems, CDSS) making and survival analysis (Sollini et al. 2020a; Handelman et al. 2018). Moreover, natural language processing might be used to extract relevant information by medical reports (Spyns 1996). Currently, the key question is how apply AI to clinical practice to ensure patients better outcomes. Many answers can be listed: accelerating the diagnostic process to deliver appropriate, effective and timely treatments, helping in facing issues related to personal skills and experiences improving the clinical workflow by automating repetitive tasks, assessing, and prioritizing normal and unhealthy cases, in order to identify those which require urgent referral. Lastly, AI dealing with “big data,” can be used to combine different data sources.

AI in medical imaging is at an early stage of development, and there are still hurdles to take related to reliability, user confidence, and adoption (Porenta 2019). Table 1 summarizes the main definitions in the field of AI.

**Table 1:** Summary of the main definitions in the field of artificial intelligence (AI)

Term (alphabetical order)	Definition
Computer-aided diagnosis and detection (CAD) systems	A technology combining elements of artificial intelligence and computer vision that analyses imaging findings to estimate the likelihood that the feature represents a specific disease process (Suzuki 2012).
Clinical decision support system (CDSS)	“A software designed to be a direct aid to clinical-decision making, in which the characteristics of an individual patient are matched to a computerized clinical knowledge base and patient-specific assessments or recommendations are then presented to the clinician for a decision” (Sim et al. 2001).
Deep learning (DL)	A specific method of machine learning that incorporates artificial neural networks in multiple layers (i.e., “deep”) to iteratively learn from data, capable of autonomously identifying highly complex patterns in large datasets (Kirienko et al. in Press).
Distributed learning	Health data network to share data that are made available to remote users by way of a query interface (Brown et al. 2010).
Intelligent system	A systems that “learns from experience and makes appropriate choices” (Poole et al. 1998).
Machine learning	A branch of artificial intelligence characterized by multiple hidden node layers that automatically learn data representations (i.e., “from experience”) by abstracting it in many ways (i.e., without being explicitly programmed) (Kirienko et al. in Press).
Natural language processing	A subfield of artificial intelligence that classify and translate text, retrieve information, generate text, and interpret human language (Davenport & Kalakota 2019).
Neural networks (NN)	“Also known as artificial NN, are networks using multiple layers of calculations to imitate the concept of how the human brain interprets and draws conclusions from information”(Handelman et al. 2018).
Holomics	The gathering of genomic, radiomic, proteomic, clinical, immunohistochemical data, and their integration in predictive or prognostic models (Gatta et al. 2020).
Radiomics	A mathematical method to extract from medical images handcrafted features (Sollini et al. 2019b).
Reinforcement learning	“A computer program receives inputs from a dynamic environment in which it must achieve a certain goal (driving or playing against an adversary). During interaction with its problem space, the program receives feedback—rewards—which it attempts to maximize” (Kirienko et al. in Press).
Semi-supervised algorithm	A machine learning method based on labeled and unlabeled input data used to learn a certain a task (van Engelen & Hoos 2020).
Statistical learning	“A vast set of tools for understanding data which learn on the basis of some aspect of the statistical structure of elements of the input, primarily their frequency, variability, distribution, and co-occurrence probability” (James et al. 2013).
Supervised algorithms	A process of an algorithm building a statistical model for predicting, or estimating, an output based on one or more inputs (training dataset) (James et al. 2013).
Texture	A general term to describe the variation of the intensity (more in general the appearance) of a surface or a volume used to quantify regional descriptors in pattern recognition (e.g., cosmology, art, and medical imaging) (Sollini et al. 2018).
Unsupervised algorithms	“A model the underlying structure or distribution in the data in order to learn more about the data” (James et al. 2013).

### Distributed learning

One of the biggest challenges for a proper application of AI within medical imaging is the requirement of a large quantity of data to train and test algorithms, especially when high variability within the population is expected. Sample sizes are often limited (or not large enough), particular in rare diseases, possibly resulting in model with a low generalizability. Multi-center studies significantly increasing the sample size as well as sample diversity may be a solution to the foregoing. However, multi-institutions research requires high standard for security and data sharing. Of note, countries, hospitals, and other stockholders follow somehow different legal, privacy, and ethical issues, making hard collaborations in health. Ideally, patients’ data is centrally shared, and the model is developed and trained on the whole dataset. An appealing alternative to the centralized approach is to distribute locally the trained model that has much lower storage requirements than the whole dataset and does not contain any individually identifiable patient information. Thus, distribution learning models across institutions—sharing model architectures and parameters—can cross the hurdles of centralized patient data preserving legal, privacy, and ethical requirements. The network, benefitting from many data provided by each individual center that contributes to an aggregated model without sharing raw data, achieves high accuracy with relative low computational resources and communication bandwidth (Vepakomma et al. 2018). However, the best method of performing such a task is still under investigation. NVIDIA in collaboration with King’s College London have recently announced the introduction of the first privacy-preserving federated learning system for medical image analysis (Suk et al. 2019), and other promising preliminary experiences of distributed learning approaches have been recently reported in medical imaging (Remedios et al. 2020; Remedios et al. 2019; Chang et al. 2018; Sheller et al. 2019).

### Statistical learning

One of the potential advantages in the use of AI as statistical tool is inherent to ML algorithms which result in a less interpretability of the models compared to “classical” statistics, high versatility, and a large variety of variables in different formats (Handelman et al. 2018). This could be particularly useful in quantitative image analysis such as radiomics. Radiomics is a field of image analysis that relies on the concept that “medical images are more than pictures to be visually interpreted,” entailing a large number of information underlying pathophysiological features and the progression of a certain disease (Gillies et al. 2016). Eventually, radiomics results in a huge number of features (from tens to thousands) that describes a lesion, a tissue or an organ. Radiomics symbolized the concept of precision medicine, in which imaged-derived biomarkers deliver timely the proper treatment for the respected patient. Imaging-derived biomarkers must be reliable and validated to speed up biomedical discoveries and to arm clinicians with new tools and knowledge that will enable personalized medicine. Nonetheless, features’ interpretability is still an issue (Ibrahim et al. 2020), and it is highly dependent from the method used for computation (conventional parameters, first order or shape and size features versus higher order, Laplacian, or wavelet features). Moreover, radiomics is—or should be—integrated with other variables known to be clinically meaningful to make a diagnosis or to predict a certain outcome. Therefore, radiomic analysis ends up with countless diverse parameters that should be used to build a model. Collectively, ML approaches—if appropriately used—could be fitter than classical statistics methods for radiomic analysis (e.g., unsupervised features selection). In addition, by combining image-derived biomarkers with demographics and clinical data, gene expression profiles, and/or other information, it is possible quantitatively and objectively support decisions making related to cancer detection and treatment (Lambin et al. 2017). Radiomics can be applied in a number of conditions including benign diseases, but the support provided by the National Cancer Institute (NCI) Quantitative Imaging Network (QIN) and by other similar initiatives promoted by the NCI Cancer Imaging Program makes oncology the main field of application until now.

### CAD system and fully automated image analysis tool

Computer-aided diagnosis and detection (CAD) system, functioning as second opinion, provides decision support to assist imagers in image interpretation. Automated detection (and segmentation) of a volume of interest is a necessary requirement for the arrangement of image-based secondary analyses used to predict a certain output (e.g., stage, outcome) as detailed in the following sections (Shen et al. 2017). However, these approaches in hybrid imaging are still in the research setting being not approved for their clinical routine applications (Tzanoukos et al. 2019; Sibille et al. 2020; Perk et al. 2018; Montgomery et al. 2007; Tzanoukos et al. 2016). The majority of FDA-approved CAD devices in oncology are used as second opinion in radiological imaging (mainly for screening).

In nuclear medicine, there are few examples such as the automated Bone Scan Index (aBSI) technology, a CE-marked and it has been available for clinical use in the United States (https://exini.com) aiming to fully quantify the metastatic bone involvement. Other approved CADs are focused on non-oncological diseases in both cardiology (i.e., coronary artery disease) and neurology (i.e., dementia).

As above-mentioned, CDSS aim to help clinicians to end up with appropriate and timely choice about patient care, to decrease errors and costs, delayed or misdiagnosis, to be compliant with patients’ management and clinical guidelines, and to ultimately ensure high quality standard for healthcare (Karami 2015). CDSS applied to images may provide alerts to inform about the appropriateness of the procedure, may suggest a priority or a diagnosis (lesion detection and/or malignancy risk).

Fully automated image analysis tools are mainly based on deep learning algorithms. These approaches aim to autonomously (i.e., without explicit human programming) identify relevant features to predict a certain outcome starting from images (Kirienko et al. in Press).

### Natural language processing (NLP)

In medicine, the main applications of NLP involve the creation, understanding, and classification of clinical notes and published research (Davenport and Kalakota 2019). NLP enables to classify and translate text, retrieve information, generate text and answering question, and finally interpret human language helping to decode meaning. Overall, NLP tools are capable to analyze unstructured medical records, suggest reports, reproduce human interactions including conversation and sentimental analysis (Davenport and Kalakota 2019). Amazon’s Alexa and Apple’s Siri accounted among the intelligent voice-driven interfaces that utilize NLP to reply or to answer to vocal suggestions or questions. In healthcare, NLP may be used to recognize and predict specific diseases based on data collected within the electronic medical records, or for example to interview a patient as in the case of chat boxes (Table 2). This capability is being explored in cardiovascular diseases, depression, and schizophrenia. In medical imaging, NLP may be used with different purposes including diagnostic surveillance and screening program, case selection within clinical trials, and quality assessment of reports in clinical practice (Pons et al. 2016). In hybrid PET/CT imaging, NLP has been used to interpret the scan and incorporate the interpretation in lexical prediction (Eyuboglu 2019). One of the main advantage of using NLP is processes’ automation which significantly reduces–or even obviates—the effort to manually review and assess a large data sets, making feasible tasks that previously were not contemplated (Pons et al. 2016). In addition, NLP may optimize clinical and diagnostic workflows by background monitoring of reporting, prioritizing, and alerting imagers or referring physicians.

**Table 2:** main chat box and corresponding website

Chatbox	website
Eva (Bots4Health)	https://chatfuel.com/bot/bots4health
OneRemission	https://keenethics.com/project-one-remission
Babylon Health	https://www.babylonhealth.com
Youper	https://www.youper.ai
Symptomate (Infermedica)	https://infermedica.com
Safedrugbot	https://www.safeinbreastfeeding.com/safedrugbot-chatbot-medical-assistant
Healthloop	https://www.getwellnetwork.com
Florence	https://florence.chat/
Your.Md	https://www.your.md
Cancer Chatbot	https://www.ihadcancer.com
Sensely virtual assistant Molly	https://www.sensely.com
Gyant	https://gyant.com
Ada Health	https://ada.com
Buoy Health (Harvard Innovation Laboratory)	https://www.buoyhealth.com

### Clinical applications of AI to oncological hybrid imaging

**Fig. 2:**
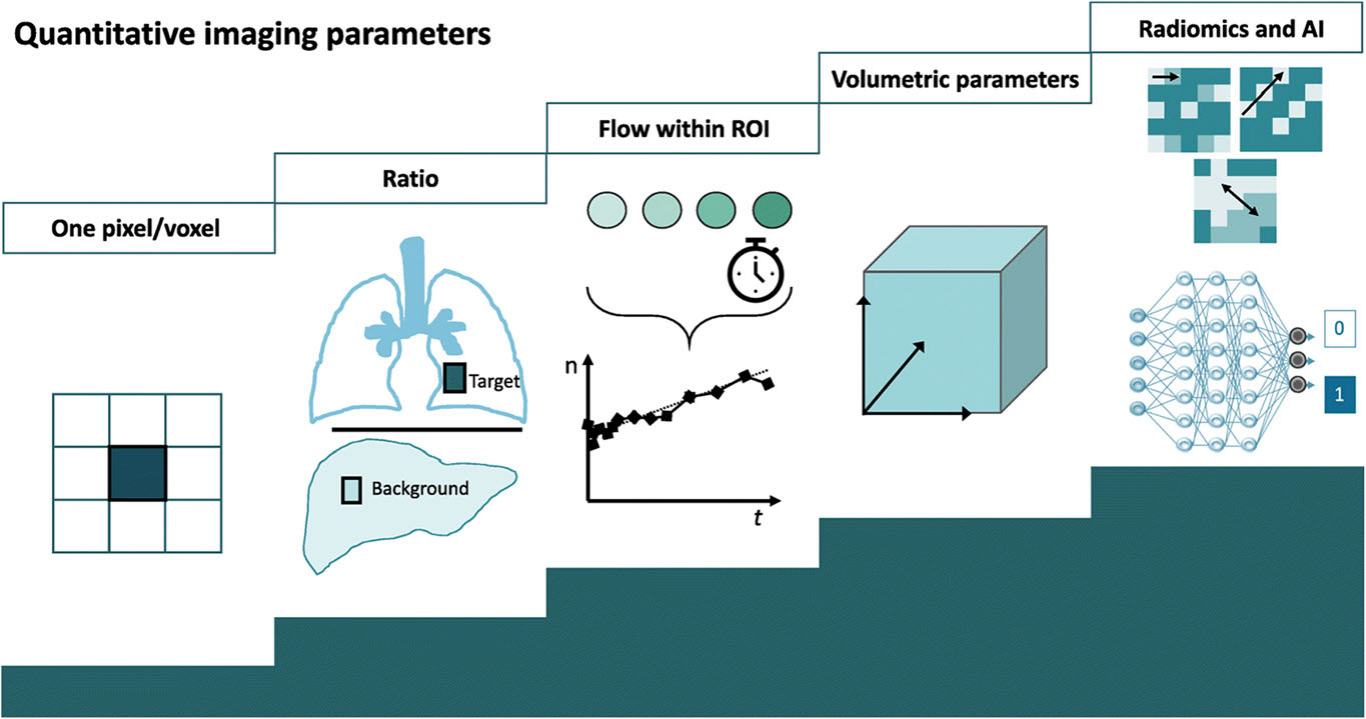
Types of quantitative approaches in imaging (reprint with permission from (Sollini et al. 2019b))

As above-mentioned, AI approaches are extremely different in terms of objectives, applications, and architectures, each tailored on the final goal. In hybrid imaging, AI-based approaches deal mainly with lesions detection and segmentation, diagnosis (benign vs malignant), staging (early vs advanced), and prognostication (favorable outcome vs poor prognosis). Algorithm input might be hand-extracted data or non-structured raw data (Ibrahim et al. 2020). Quantification to obtain imaging biomarkers in nuclear medicine has evolved with a progressive increasing degree of complexity (Fig. 2). Extraction and analysis of quantitative data from nuclear imaging data is a standard procedure. Conventional semiquantitative parameters such as standard uptake value (SUV), metabolic tumor volume (MTV), total lesion glycolysis (TLG), and absolute quantitative number (i.e., glomerular filtration rate, coronary blood flow) are obtained applying proper image correction and reconstruction algorithms combined with tracer kinetic modeling techniques (Sollini et al. 2019b). Nevertheless, the use of advanced—hybrid—image analysis (i.e., radiomics and machine learning algorithms), changes the field of in vivo disease characterization (Gillies et al. 2016). Radiomic features provide information relating to space and texture on the grayscale patterns and describe the relationship between pixels or voxels within an image. Manual extracted features can be modeled into AI-based systems that can improve personalized medicine supporting diagnosis and treatment guidance (Parekh and Jacobs 2016; Coronary flow reserve and the J curve 1988; Ibrahim et al. 2019). Radiomic PET and PET/CT analysis has been used to widen visual assessment and conventional semiquantitative parameters (Thie 2004; Chicklore et al. 2013; Leijenaar et al. 2013; Cheebsumon et al. 2012; Tixier et al. 2014; Nair et al. 2012; Oliver et al. 2015; Vallières et al. 2015; Yoon et al. 2015; Ypsilantis et al. 2015; Antunes et al. 2016; Bailly et al. 2016; Desseroit et al. 2016; Grootjans et al. 2016; Lian et al. 2016; van Velden et al. 2016). Leijenaar et al. showed a high test-retest stability for radiomic features derived from PET images, which outperformed inter-observer’s variability in repeated PET examinations and exhibited higher prognostic capability (Leijenaar et al. 2013). Several studies are going beyond the use of hybrid imaging, combine different data sources and empower models’ predictive capabilities in the so-called *“Holomics”* approach (Way et al. 2017; Emblem et al. 2015; Zhang et al. 2016). This integrated “front end” approach is crucial to understand complex diseases where several players (e.g., pathogen, microenvironment, host defense) act and interact with many suppressive/facilitating mechanisms at different levels—molecular, cellular, and organism (Holzinger et al. 2019). Indeed, for example, this approach empowered recurrence risk prediction in stage I primary non-small cell lung cancer patients compared to genomic biomarkers (Emaminejad et al. 2016).

Among the possible clinical scenario, lung cancer represents the most extensively studied. Therefore, we will use it as a model to show the possible clinical implication of AI.

### Lung cancer

Lung is the second most frequent tumor for incidence in Europe (12.1%), but the first one for mortality (20.9%) (ECIS 2020). It is surely the most extensively studied and characterized malignancies Moreover, lung cancer, being still distinguished by a number of unmet clinical needs, may serve as an exemplary scenario to test new approaches. In the field of lung imaging, CNNs have been tested in the following clinical questions: nodule detection and segmentation from CT images, cancer risk assessment in patients with lung nodules, staging, and prognosis.

### Tumor detection and segmentation

Very recently, an innovative end-to-end CNN-based method to detect, contour and extract metabolic information in total-body PET/CT has been developed in lymphoma and tested in both lymphoma and advanced NSCLC within a multi-center trial with very promising results (93% sensitivity in the NSCLC cohort) (Jemaa et al. 2020). Other approaches have been explored to detect lung tumor in PET imaging. The multi-scale Mask Region-Based Convolutional Neural Network resulted highly effective in detecting lung tumor, and classifying healthy chest pattern, consequently reducing misdiagnosis (Zhang et al. 2019). Moreover, deep learning has been explored in reduced and ultralow dose PET (PET_10%_ and PET_3.3%_, respectively) to detect lung tumor. The algorithm trained with ultralow dose PET_3.3%_ loose in terms of both sensitivity and specificity compared to standard dose (sensitivity of 91% and 96%, specificity of 94% and 98% for ultralow dose PET_3.3%_ and PET_100%_, respectively). Nonetheless, the AUCs were almost comparable for standard dose images, reduced dose PET_10%_, and PET_3.3%_ reconstruction (0.99, 0.98, and 0.97), respectively (Schwyzer et al. 2018). However, the detection rate of AI-based algorithms in PET/CT images seems to be affected by tumor stage (i.e., the more advanced stage, the lower detection rate). Particularly, AI-based algorithms performance has been reported to be weak in the T3 category (detection rate of about 30%), and poor in the T4 one (detection rate of approximately 9%), being the pleura contact and/or involvement the main determinant for misdetection. Similarly, T1 tumors and tumors without pleural contact were excellently segmented. Conversely, the algorithm systematically underestimated volumes of sizable tumors. Accordingly, efforts should focus on facilitating segmentation of all tumor types and sizes to bridge the gap between CAD applications for lung cancer screening and staging (Weikert et al. 2019).

Fully automated segmentation methods in PET have been proposed, using fuzzy random walk (Soufi et al. 2017) or mutual information of CT and PET to identify NSCLC (Weikert et al. 2019; Bug et al. 2019). U-Net, one of the mostly used CNN architectures for image contouring, has shown to be able to segment pulmonary parenchyma (Ait Skourt et al. 2018), and relatively small tumors (1.83 cm^2^) resulting reproducible across different scanners (dice scores of 74%), relatively uninfluenced by the partial volume effect, and effectively trained with limited data (30 patients yielded a dices score of 70%) (Leung et al. 2020). The use of information derived from both CT and PET components of hybrid imaging, being complementary providing data on morphology and metabolism, improved U-net performance in segmenting lung tumor (average dice scores of 82% and 80% for the training and test datasets, respectively) (Wang et al. 2017a).

### Prediction of histology and tumor stage

Full characterization and prediction of three-dimensional image-based histology of the lesion is a very exciting and challenging area of research. Different algorithms (naïve Bayes’ classifier, random forest, LDA) have been tested to predict histology with good results, mainly using radiomic features as input with CT and/or PET images. Kirienko et al. modeled CT and PET radiomic features into machine learning algorithms to firstly differentiate primary lung cancer from lung metastases, and secondly to subtype primary lung tumor (Kirienko et al. 2018a). PET-derived features outperformed CT ones in classifying lung lesions as primary tumors and metastases (area under the curve—AUC of 0.91 ± 0.03 and 0.70 ± 0.04 for PET and CT, respectively). More disappointing results were achieved in differentiating primary subgroups, especially in the case of CT (AUC = 0.57–0.70) (Kirienko et al. 2018a). Almost comparable results were obtained using naïve Bayes’ classifier to predict histopathology from CT-extracted radiomic features selected by relief-based algorithm AUC = 0.72 in the validation cohort) (Kirienko et al. 2018a; Wu et al. 2016) and artificial neural networks (Ferreira Junior et al. 2018). Hyun et al. combined clinicopathological data (age, sex, tumor size, and smoking status), conventional PET parameters, and radiomic features to predict the histological subtype (adenocarcinoma versus squamous cell carcinoma) building different models (Hyun et al. 2019). Sex, total lesion glycolysis, gray-level zone length non-uniformity, and gray-level non-uniformity for zone resulted the best predictors of the histological subtype, (AUC = 0.86 and 0.85 for the logistic regression and neural network models, respectively).

CT-extracted radiomic features have been also related to NSCLC tumor stage (Ganeshan et al. 2010) and to lung adenocarcinoma micropapillary patterns (Song et al. 2017). Machine learning models combining clinical variables, visual qualitative CT features, and SUV_max_ predicted nodal involvement in early T-stage NSCLC with high accuracy (AUC = 0.78–0.90) (Wu et al. 2020). Liao et al. proposed a promising 3D deep neural network to automatically diagnose tumor in patients with lung nodules (AUC = 0.90 and 0.87 for the training and test sets, respectively) (Liao et al. 2019). Similarly, PET/CT-based CNN has proven to be promising in staging primary lung lesion as T1–T2 or T2–T3 (Kirienko et al. 2018b).

The value of PET/CT-based CNN has been also investigated and compared to classical machine learning methods to predict mediastinal nodal NSCLC involvement. The CNNs took cropped PET/CT images as input, while conventional and radiomic features were the input for classical machine learning methods. Moreover, AI-based approaches were compared to physicians’ performance. Conventional parameters outperformed radiomics for the classical machine learning methods, while performance of CNN and the best classical method were not significantly different. Interestingly, all the AI-based algorithms had higher sensitivities but lower specificities than physicians (Wang et al. 2017b). A similar experience with a supervised machine learning approach has been reported in naïve newly diagnosed NSCLC. The algorithm predicted with a good diagnostic accuracy the nodal involvement (0.80 ± 0.17), but with disappointing result in the presence of distant metastases (0.63 ± 0.05) (Tau et al. 2020).

### Prediction of mutational status and molecular therapies targets

Mutational status and molecular therapies targets impact on the NCSCL patients clinical workflow, especially since new targeted drugs got FDA approval (Sun et al. 2018). Accordingly, genomic analysis is growing to be routinely performed in lung cancer, and interest has grown on the chance to predict tumor mutational status and molecular therapies targeted by radiomics. Halpenny et al., investigating the predictive value of CT radiomics for ALK rearrangements, were the pioneers of radiogenomics of (Halpenny et al. 2014). Early on, several studies selected clinically relevant mutations such as ALK (Yoon et al. 2015), EGFR (Caramella et al. 2015; Aerts et al. 2016), and KRAS (De Jong et al. 2016), with different approaches (correlative studies versus predictive models) (Weiss et al. 2014). More recently, some CNN-based approaches (CT images as input) have been developed to predict EGFR mutation in stage I–IV adenocarcinoma with results ranging from good to excellent (AUC = 0.75–0.84), (Zhang et al. 2018; Wang et al. 2019a; Koyasu et al. 2019; Jiang et al. 2019). Interestingly, the implementation of CNN-based models with clinicopathological variables improved models’ performance (Koyasu et al. 2019; Jiang et al. 2019). AI-based approaches, outperformed visual analysis and radiomics (AUCs 0.81 versus 0.64–0.74) (Li et al. 2018; Wang et al. 2019b). However, CNN-based approaches may be combined with radiomics features. Again, the addition of clinical parameters (sex, smoking) improved model’s performance (AUC 0.83 vs 0.81) (Koyasu et al. 2019). Gevaert et al. and Nair et al. correlated PET/CT features to metagenes (i.e., aggregated gene expression patterns) (Gevaert et al. 2012; Nair et al. 2014). The majority of the studies focused on hybrid PET/CT aimed to predict EGFR status by radiogenomic signatures using machine learning approaches (Koyasu et al. 2019; Li et al. 2019) Koyasu et al. compared random forest and gradient tree boosting classifiers used single type or multiple types of imaging features. The best AUC was achieved by using the gradient tree boosting classifier with Bayesian optimization multiple types of imaging features (AUC = 0.66) (Koyasu et al. 2019). Jiang et al. assessed EGFR gene mutation status in 80 stage I–II NCSCL patients by using PET/CT radiomic features and CT qualitative features. The performance of the support vector machine model resulted outstanding (AUC = 0.95) (Jiang et al. 2019). Li et al. compared hybrid PET/CT radiomic signature to single PET or CT radiomic fingerprint, and to conventional PET parameters. As expected, the signature extracted from both PET and CT images outperformed the “single imaging modality” signatures in discriminating between mutant-type and wild-type EGFR cases (AUC = 0.80) (Li et al. 2019). Some other studies aimed to predict the expression of PD-1/PD-L1 to select patients for immune-checkpoint inhibitors (Jiang et al. 2020; Yoon et al. 2020).

### Prediction of treatment response and outcome

AI has been used to predict lung cancer outcome (i.e., local/distant control) and survival in variously treated patients (radio-, and/or chemotherapy, and/or targeted molecular therapy, and/or immunotherapy).

Huang et al. proposed a machine learning algorithm to identify the optimum prognosis index for brain metastases in a large cohort of patients with different tumors (446 training and 254 testing), mainly NSCLC (*n* = 635) (Huang et al. 2019). Seven clinical and qualitative features (age, Karnofsky Performance Status, extracranial metastases, primary tumor control, number of lesions, maximum lesion volume, chemotherapy administration) and seven supervised machine-learning algorithms were used to predict patients’ prognosis. The mutual information and rough set with particle swarm optimization (MIRSPSO) performance was far higher than that of conventional statistic methods achieving the highest accuracy in survival prediction (AUC = 0.978 ± 0.06).

A multi-objective Bayesian network was developed to predict both local control and radiation pneumonitis in a prospective cohort of 118 NSCLC. Selected features included single nucleotide polymorphisms, micro RNAs, pre-treatment cytokines, pre-treatment PET radiomics, lung, and tumor gEUDs. Interestingly, model’s performance improved (AUC from 0.80 to 0.85) when adding additional during-treatment information (additional two SNPs, changes in one cytokine, and two radiomics PET image features) (Luo et al. 2018).

Supervised principal component analysis has been used to select several combinations of PET/CT features and predict recurrence after SBRT (Oikonomou et al. 2018; Dissaux et al. 2020).

Predictive models based on CT (Huynh et al. 2016) and PET/CT (Oikonomou et al. 2018) have been built to assess the risk of distant metastases in NSCLC cancer patients treated with SBRT. Coroller et al. found the combination between radiomic features (wavelet HHL skewness, GLCM cluster shade, Laplacian of Gaussian 5 mm 2D skewness,) and clinical data predictive for both distant metastasis and survival in locally advanced adenocarcinoma (Coroller et al. 2015). Random forest classifier exhibited good performance in predicting the risk of recurrence in stage I–III NSCLC patients surgically treated (Ahn et al. 2019).

Interestingly, also radiomic features extracted from the peritumoral area seem hold predictive information as demonstrated by Hao et al. (2018) and Khorrami et al. (2019).

Hawkins et al. selected five CT-based features using the relief-based approach and trained a decision trees classifier to predict survival with good accuracy (78%) in the leave-one-out cross validation (Hawkins et al. 2014). Moreover, the SumMean PET-derived radiomic feature extracted from pretreatment scans has been identified by LASSO as an independent predictor of overall survival in lung cancer (Ohri et al. 2016). Deep learning algorithms applied to time series CT examinations significantly predicted survival and cancer-specific outcomes (Xu et al. 2019; Paul et al. 2016). Deep learning has also been compared to the standard of care CT to retrospectively assess the mortality risk in about 1200 NSCLC patients from 7 institutions. The proposed deep learning approach resulted able to predict survival (> or < 2 years) in patients treated with radiotherapy or surgery (Lafata et al. 2019). Similarly, a model based on changes in intensity in intra-tumor partitions was able to predict 2-year overall survival outperforming radiomics (Astaraki et al. 2019). Amsterdam et al. combined deep learning algorithm to structural causal model to achieve unbiased individual prognosis predictions from CT images (van Amsterdam et al. 2019). Interestingly, the U-Net segmentation algorithm (CNN-based architecture) identified a large number of survival-related PET and CT features with a remarkable prognostic value and strongly correlated with 2- and 5-year overall survival and disease-specific survivals. Meaningfully, there was a spatial match between metastasis/recurrence and the regions where the U-Net algorithm predicted higher likelihoods of death (Baek et al. 2019).

Deep learning has also been applied to automatically measure and correlate anthropometric parameters (subcutaneous/visceral adipose tissue and muscular body mass) from low-dose CT images to survival in NSCLC patients who underwent pretherapy PET/CT. The fully automated task made anthropometric CT-derived parameters clinically usable, being manual computation time-consuming. Whole-body CT-derived anthropometrics result was associated with progression-free survival and overall survival (Blanc-Durand et al. 2020).

Recently, Bayesian network and SVM have been compared to predict 2-year survival in NSCLC treated with EBRT. Gross tumor volume size, performance status, and number of positive lymph nodes on PET were identified as prognostic factors by machine learning approaches. Both models had similar performance when considering patients with complete data set, conversely, as expected Bayesian network which has a natural ability to reason under uncertainty, resulted more accurate than SVM in handling missing data (AUCs = 0.70–0.77 and 0.68–071, respectively) (Jayasurya et al. 2010). AI-based approaches have been explored with promising results also to develop automated radiation adaptation protocols to maximize tumor local control and reduced rates of radiation pneumonitis (Tseng et al. 2017).

Radiogenomics has been used to build prognostication models (Grossmann et al. 2017). Emaminejad et al. found that the combination of radiomics and genomics outperformed the single data source in predicting survival (AUC = 0.84 versus 0.78 and 0.78, respectively) (Emaminejad et al. 2016). AI-based approaches alone or combined with clinical parameters have been tested to predict EGFR mutational status with good performance (AUCs 0.75–0.84) (Li et al. 2018; Wang et al. 2019b; Xiong et al. 2018; Zhao et al. 2019), outperforming visual analysis and radiomics (AUCs 0.81 versus 0.64–0.74) (Li et al. 2018; Wang et al. 2019b).

## Discussion

**Fig. 3:**
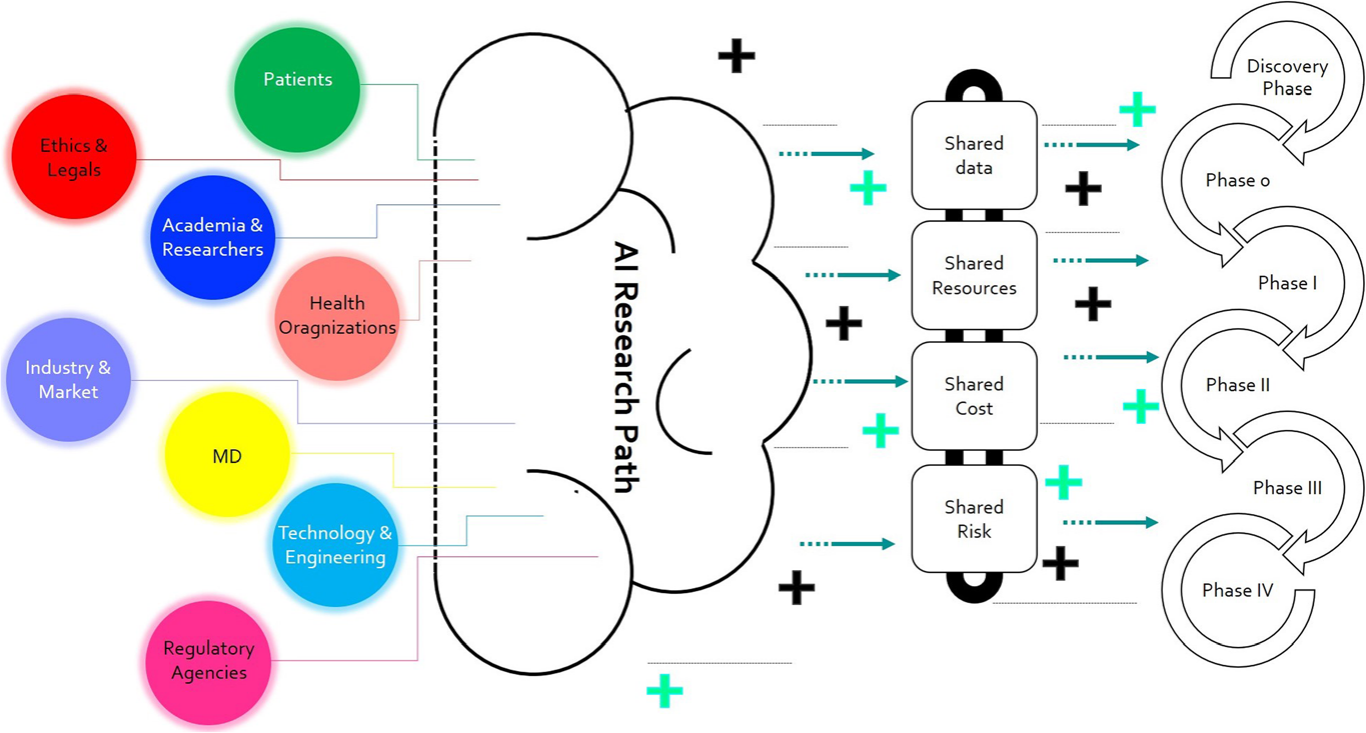
Multi-dimensional interactions among stakeholders involved in the AI research path

It should be taken into account that “data are more important than hardware or software in determining the success of AI applications” (Porenta 2019). Interest and research in medical imaging AI application has been exploded in recent years; however, before the widespread implementation and integration of AI into clinical practice, several hurdles need to be overcome since its scientific evidence is still limited (Porenta 2019). Among the main important AI-related issues, we can find appropriate validation methods, the development of effective data sharing platforms, ethics and regulatory aspects, the settle up of adequate educational program, and proper strategy to facilitate the interaction among the multiple stakeholders, i.e., partnerships among academia, researchers, healthcare and patients organizations, charities, and industry (Fig. 3).

### Standard and quality

To successfully implement AI in the real-world clinical applications and to guide clinical decisions, high standards are needed for model development (image protocol), training (high standard for image labeling, features extraction, and reference standard), and testing such as methods reproducibility, feature reduction, selection, and validation, biological/clinical meaning, performance index, high level of evidence, being all necessary prerequisites to make results reliable, explainable, and interpretable (Sollini et al. 2020a; Porenta 2019; Kocak et al. 2020). High standard quality should be guaranteed in medical research regardless of the index test. Therefore, quality assessment is mandatory to provide interpretable, transparent, reproducible, and informative results even in AI-based studies (Ninatti et al. 2020). Lambin et al. developed a score system, including radiomics quality score (RQS), to inform on the validity and completeness of radiomics studies (Lambin et al. 2017). The RQS, inspired to the Transparent Reporting of a multi-variable prediction model for Individual Prognosis OR Diagnosis (TRIPOD) initiative, includes 16 topics (Collins et al. 2015). Ninatti et al. proposed and demonstrated the feasibility of a modified TRIPOD checklist (specific items scarcely or unfit for AI-based investigations were adapted or ignored) to assess AI-based models’ quality (Ninatti et al. 2020). In this regard, an “ad hoc” TRIPOD statement has been recently proposed (Collins and Moons 2019). Moreover, data sharing now is possible due to a number of available repositories and should be promoted to confirm the reproducibility, robustness, and reusability of the research results as recommended by the FAIR (findability, accessibility, interoperability, and reusability) principles (Wilkinson et al. 2016).

### Sample size, overfitting, and validation

As above-mentioned, validation is an essential requirement for reliability of AI applications. Validation may handle with a number of approaches which essentially differ for the modality of collecting and splitting data. New collected data or separate dataset are among the most robust. The Train/Test Split is another common approach which consists in separating some data before the development of the machine learning model. These data will be used only for the validation step. When the sample size is limited, internal validation (e.g., K-Fold cross-validation) is a simple solution. This approach goes through the well-defined model’s development pipeline (data normalization, selection, parameters optimization). Thereafter, some data is refrained for validation, using the rest to train the model and the left-out validation folds to test the prediction. This process is repeated several times, by excluding different parts of the data for validation until all the data is used. The mean of each validation folds classification performances serves as overall model’s performance. Theoretically, cross-validation should more accurately estimate the sample, compared to other internal validation approaches even if can some data used to validate the prediction might overlap with data used for the model’s training (Vabalas et al. 2019). Accordingly, CV used for model development and model evaluation should be different (Stone 1974) otherwise overestimation performance are expected (Varma and Simon 2006) (Vabalas et al. 2019). Vice versa, the nested CV approach revealed unbiased performance estimates. Therefore, the complete separation between testing and training seems to be sufficient to achieve unbiased performance estimates, regardless of sample size. When only part of the model is developed, feature selection is more relevant than parameter optimization to prevent overfitting. Other factors including data dimensionality, hyper-parameter space, number of CV folds, and data discriminability influence bias and should be taken into account. Moreover, the trade-off between bias and variance is crucial in machine learning—regardless of the approach used for validation—to develop models that generalize well (Kocak et al. 2020).

### Data mining and infrastructures

Data collection and data exchange are challenging. Benchmarking trials on Open datasets providing accessible data, result an awesome source, especially to gain robustness in radiomic and AI-based benchmarking research which typically require access to large numbers. Nonetheless, data repository creation is challenging and uncommon outside research programs, since it requires multi-sources privacy-preserving platforms with a high computational power expected to proficiently process raw data. Recently, the Genomic Research and Innovation Network (GRIN) initiative has recently developed an infrastructure armed with distributed learning technology, to integrate multi-sources data, harmonize biobanking procedures, and transfer agreements to ultimately create a scalable genomic research network (Mandl et al. 2020). This collaboration perfectly illustrates the feasibility of a multi-dimensional infrastructure to manage, analyze, and integrate data coming from different domains.

### Ethical issues, data protection, regulation, and privacy

The majority of the ethical concerns related to healthcare applications of AI, are summarized into the “fairness, accountability, and transparency (FAT) paradigm of AI ethics” (Hagendorff 2020). Explainability and interpretability of AI methods (and output) are still an issue since the impracticability of going backwards from the output to the input (Ninatti et al. 2020). Moreover, AI-based tools in healthcare dealing with sensitive information, also raises concerns about data protection and privacy. The introduction of distributed learning (Deist et al. 2020), will promote multi-institutional collaboration, while preserving privacy constraints. Indeed, efficient algorithms to be generalizable require large numbers and a tremendous variability within the population, raising concerns around consent, data anonymization, and de-identification. Some initiatives have been started in this regard (Zittrain 2020; Allen and Chan 2020). AI-based software follow the same regulation of medical devices (Pesapane et al. 2018) but their specific feature (i.e., learning and adapting to new data in real time to increase performance) has raised concerns about the suitability of traditional medical device regulatory pathways resulting in a FDA’s proposal to modify this regulatory framework (FDA 2019). Until 8 August 2019, more than 100 comments were submitted in response to the proposed regulatory framework, but they lacked in scientific evidence (only 15 comments cited at least 1 paper—systematic review/meta-analysis in only 5 cases—published in an academic journal) while resulting certainly related to parties with financial ties in about two third of the cases (Smith et al. 2020). Obviously, financial ties represents a conflict of interest and, as such, it should also be disclosed in the regulatory pathways as typically occurs in medical science, since it may, even not necessarily, lead to biased commenting (Smith et al. 2020). Disclosure is an important first step in improving transparency and reducing privacy concerns especially when it deals to sensitive data such as in healthcare even if it will not necessarily alter the interpretation of information (Pesapane et al. 2018; Smith et al. 2020).

### Education and acceptance

Patient engagement and adherence is crucial for good health outcomes. In 2003, the World Health Organization stated that “increasing medication adherence might have a far greater impact on the health of the population than any improvement in specific medical treatments” (Chaudri 2004). Barriers related to non-adherence (i.e., patients’ emotions, patients’ intention to not take medications, emotional distance from health care providers, social, and cultural beliefs insufficient information) has recently become more apparent being increasingly addressed by big data and AI. Recently, results of a patient survey on the implementation of AI in radiology demonstrated that they are fairly disappointing when it comes to their trust in AI in replacing imagers within the classical diagnostic interpretation framework (accuracy, communication, and confidentiality). This was due to the understanding how their data are processed (acquired, interpreted and communicated) and how AI approaches will improve efficacy of radiology, and that they definitely preferred a doctor-patient relationship over AI-based communication (Ongena et al. 2020). Overall, there is growing emphasis on using machine learning to drive personalized interventions along the care continuum (Volpp and Mohta 2016), but the future is still uncertain and there is not a definite answer to the question “Will be imagers outgunned by “intelligent” algorithms?” (https://www.radiologybusiness.com/topics/artificial-intelligence/wait-will-ai-replace-radiologists-after-all^2020^). Notwithstanding this question has been outdated by the more politically correct motto that “imagers using AI will replace those who don’t” (Hustinx 2019), ungraduated medical students are probably more aware on AI medical potentiality and business opportunities, than nowadays imagers rather afraid of turf losses (Pinto Dos Santos et al. 2019; van Hoek et al. 2019). What we—as physicians and academia—should be familiar with is that to incorporate AI into daily practice, it is necessary to provide users a basic knowledge in artificial intelligence, well understanding both its pros and cons. The creation of the educational resources will require the cooperation of multiple national and international societies as well as academia and industries providing both the resources for the education of current practicing imagers and users and the development of a standard curriculum for the future generations (Kirienko et al. in Press; Kocak et al. 2020; Hustinx 2019). In this regard, specific initiatives aimed to confer upon imagers specific proficiency and skills on AI have started and promoted (Artificial Intelligence in Healthcare 2020; ESMIT Autumn School 2020; AI For Medicine 2020; AI resources and training 2020). However, artificial intelligence in medicine in not limited to image analysis, but it deals with big data, nanotechnologies, robotics, 3D printing, and bio-prosthesis, affecting other fields of expertise and specialties such as surgery, orthopedics, and emergency. The MEDTEC school is a recently launched degree program aimed to train professionals who will be able to apply medical and technological know-how as a means of providing innovative and quality medicine (https://www.hunimed.eu/course/medtec-school/2020).

### Interdisciplinarity

Interdisciplinarity, affecting research results and progresses, has been claimed as an imperative condition for discovery and innovation in different domains, affect, including advanced image analysis (Sollini et al. 2020a). Radiomic and AI (i.e., image mining) (Sollini et al. 2019a) research benefits from an interdisciplinary attitude. Overall, the chance to exchange knowledge between experts in different fields (physicians and imagers, physicists and statisticians, biologists, informatics, engineers, and data mining) remarkably impacts on methodology (i.e., quality) and confers robustness to results and ultimately promoting image mining toward clinical practice (Sollini et al. 2020b).

## Conclusions

Some of the major changes in medical imaging will come from the application of AI to workflow and protocols, eventually leading to improved patient management and quality of life. By AI, several time-consuming tasks should be automatized. Further, accurate detection and interpretation of imaging findings to better classify and quantitatively characterize local and diffuse disease can be achieved. Machine learning algorithms and neural networks will permit sophisticated analysis resulting not only in major improvements in disease characterization through imaging, but also in the integration of multiple-omics data (i.e., derived from pathology, genomic, proteomics, and demographics) for multi-dimensional disease featuring. Nevertheless, to accelerate the transition from theory to practice, it is necessary to launch a sustainable development plan which considers the multi-dimensional interactions between professionals, technology, industry, markets, policy, culture, and civil society lead by a mindset which will allow talents to thrive.

## Abbreviations

AI: Artificial intelligence
aBSI: Automated Bone Scan Index
ACR: The American College of Radiology
AUC: Area under the curve
CAD: Computer-aided diagnosis and detection
CDSS: Clinical Decision Support Systems
CNNs: Currently, convolutional neural networks
CT: Computed tomography
DL: Deep learning
EANM: European Association of Nuclear Medicine
ESR: European Society of Radiology
GLCM: Gray-level co-occurrence matrix
GRIN: Genomic Research and Innovation Network
MIRSPSO: Mutual information and rough set with particle swarm optimization
MTV: Values metabolic tumor volume
NCI: National Cancer Institute
NLP: Natural language processing
NN: Neural networks
PET: Positron emission tomography
QIN: Quantitative Imaging Network
RQS: Radiomics quality score
SUV: Standard uptake value
TLG: Total lesion glycolysis
TRIPOD: Transparent Reporting of a multivariable prediction model for Individual Prognosis Or Diagnosis
VAT: Visceral adipose tissue

## References

[CR1] Aerts HJ, Grossmann P, Tan Y, Oxnard GR, Rizvi N, Schwartz LH (2016). Defining a radiomic response phenotype: a pilot study using targeted therapy in NSCLC. Sci Rep.

[CR2] Ahn HK, Lee H, Kim SG, Hyun SH (2019) Pre-treatment ^18^F-FDG PET-based radiomics predict survival in resected non-small cell lung cancer. Clin Radiol 74(6):467–473 10.1016/j.crad.2019.02.008 30898382

[CR3] AI For Medicine. https://www.deeplearning.ai/ai-for-medicine/. Accessed on 30 Oct 2020

[CR4] AI resources and training. https://www.rsna.org/en/education/ai-resources-and-training. Accessed on 30 Oct 2020

[CR5] Ait Skourt B, El Hassani A, Majda A (2018). Lung CT image segmentation using deep neural networks. Procedia Computer Science.

[CR6] Allen G, Chan T. Artificial intelligence and national security. https://www.belfercenter.org/publication/artificial-intelligence-and-national-security. Accessed on 26 Oct 2020

[CR7] Antunes J, Viswanath S, Rusu M, Valls L, Hoimes C, Avril N (2016). Radiomics analysis on FLT-PET/MRI for characterization of early treatment response in renal cell carcinoma: a proof-of-concept study. Transl Oncol.

[CR8] Artificial Intelligence in Healthcare. https://online.stanford.edu/programs/artificial-intelligence-healthcare. Accessed on 30 Oct 2020

[CR9] Astaraki M, Wang C, Buizza G, Toma-Dasu I, Lazzeroni M, Smedby Ö (2019). Early survival prediction in non-small cell lung cancer from PET/CT images using an intra-tumor partitioning method. Phys Med.

[CR10] Baek S, He Y, Allen BG, Buatti JM, Smith BJ, Tong L (2019). Deep segmentation networks predict survival of non-small cell lung cancer. Sci Rep.

[CR11] Bailly C, Bodet-Milin C, Couespel S, Necib H, Kraeber-Bodéré F, Ansquer C (2016). Revisiting the robustness of PET-based textural features in the context of multi-centric trials. PLoS One.

[CR12] Blanc-Durand P, Campedel L, Mule S, Jegou S, Luciani A, Pigneur F (2020). Prognostic value of anthropometric measures extracted from whole-body CT using deep learning in patients with non-small-cell lung cancer. Eur Radiol.

[CR13] Brown JS, Holmes JH, Shah K, Hall K, Lazarus R, Platt R (2010). Distributed health data networks: a practical and preferred approach to multi-institutional evaluations of comparative effectiveness, safety, and quality of care. Med Care.

[CR14] Bug D, Feuerhake F, Oswald E, Schüler J, Merhof D (2019). Semi-automated analysis of digital whole slides from humanized lung-cancer xenograft models for checkpoint inhibitor response prediction. Oncotarget..

[CR15] Caramella C, Bluthgen MV, Rosellini S, Leduc C, Facchinetti F, Haspinger E (2015). 3133 Prognostic value of texture analysis and correlation with molecular profile in EGFR mutated/ALK rearranged advanced non-small cell lung cancer (NSCLC). Eur J Cancer.

[CR16] Chang K, Balachandar N, Lam C, Yi D, Brown J, Beers A (2018). Distributed deep learning networks among institutions for medical imaging. J Am Med Inform Assoc.

[CR17] Chartrand G, Cheng PM, Vorontsov E, Drozdzal M, Turcotte S, Pal CJ (2017). Deep learning: a primer for radiologists. Radiographics..

[CR18] Chaudri NA (2004). Adherence to Long-term Therapies Evidence for Action. Ann Saudi Med.

[CR19] Cheebsumon P, Boellaard R, de Ruysscher D, van Elmpt W, van Baardwijk A, Yaqub M (2012). Assessment of tumour size in PET/CT lung cancer studies: PET- and CT-based methods compared to pathology. EJNMMI Res.

[CR20] Chicklore S, Goh V, Siddique M, Roy A, Marsden PK, Cook GJ (2013) Quantifying tumour heterogeneity in ^18^F-FDG PET/CT imaging by texture analysis. Eur J Nucl Med Mol Imaging 40(1):133–140 10.1007/s00259-012-2247-0 23064544

[CR21] Collins GS, Moons KGM (2019). Reporting of artificial intelligence prediction models. Lancet..

[CR22] Collins GS, Reitsma JB, Altman DG, Moons KG (2015). Transparent reporting of a multivariable prediction model for individual prognosis or diagnosis (TRIPOD): the TRIPOD statement. BMJ..

[CR23] Coroller TP, Grossmann P, Hou Y, Rios Velazquez E, Leijenaar RT, Hermann G (2015). CT-based radiomic signature predicts distant metastasis in lung adenocarcinoma. Radiother Oncol.

[CR24] Coronary flow reserve and the J curve (1988). BMJ.

[CR25] Davenport T, Kalakota R (2019). The potential for artificial intelligence in healthcare. Future Healthc J.

[CR26] De Jong EEC, Van Elmpt W, Hendriks LEL, Leijenaar RTH, Dingemans AMC, Lambin P (2016). OC-0609: Radiomic CT features for evaluation of EGFR and KRAS mutation status in patients with advanced NSCLC. Radiother Oncol.

[CR27] Deist TM, Dankers FJWM, Ojha P, Scott Marshall M, Janssen T, Faivre-Finn C (2020). Distributed learning on 20 000+ lung cancer patients - the personal health train. Radiother Oncol.

[CR28] Desseroit MC, Visvikis D, Tixier F, Majdoub M, Perdrisot R, Guillevin R et al (2016) Development of a nomogram combining clinical staging with ^18^F-FDG PET/CT image features in non-small-cell lung cancer stage I-III. Eur J Nucl Med Mol Imaging 43(8):1477–1485 10.1007/s00259-016-3325-5 PMC5409954 26896298

[CR29] Dissaux G, Visvikis D, Da-Ano R, Pradier O, Chajon E, Barillot I (2020). Pre-treatment (18)F-FDG PET/CT Radiomics predict local recurrence in patients treated with stereotactic radiotherapy for early-stage non-small cell lung cancer: a multicentric study. J Nucl Med.

[CR30] ECIS. European Cancer Information System. https://ecis.jrc.ec.europa.eu. Accessed on 28 Aug 2020

[CR31] Emaminejad N, Qian W, Guan Y, Tan M, Qiu Y, Liu H (2016). Fusion of quantitative image and genomic biomarkers to improve prognosis assessment of early stage lung cancer patients. IEEE Trans Biomed Eng.

[CR32] Emblem KE, Pinho MC, Zöllner FG, Due-Tonnessen P, Hald JK, Schad LR (2015). A generic support vector machine model for preoperative glioma survival associations. Radiology..

[CR33] ESMIT Autumn School (2020) https://www.eanm.org/esmit/level-2/esmit-autumn-school-2020-3/. Accessed on 30 Oct 2020

[CR34] Eyuboglu E (2019). On the automatic generation of FDG-PET-CT reports.

[CR35] FDA (2019). Proposed regulatory framework for modifications to artificial intelligence/machine learning (ai/ML)-based software as a medical device (SaMD) - discussion paper and request for feedback.

[CR36] Ferreira Junior JR, Koenigkam-Santos M, Cipriano FEG, Fabro AT, Azevedo-Marques PM (2018). Radiomics-based features for pattern recognition of lung cancer histopathology and metastases. Comput Methods Prog Biomed.

[CR37] Ganeshan B, Abaleke S, Young RC, Chatwin CR, Miles KA (2010). Texture analysis of non-small cell lung cancer on unenhanced computed tomography: initial evidence for a relationship with tumour glucose metabolism and stage. Cancer Imaging.

[CR38] Gatta R, Depeursinge A, Ratib O, Michielin O, Leimgruber A (2020). Integrating radiomics into holomics for personalised oncology: from algorithms to bedside. Eur Radiol Exp.

[CR39] Gevaert O, Xu J, Hoang CD, Leung AN, Xu Y, Quon A (2012). Non-small cell lung cancer: identifying prognostic imaging biomarkers by leveraging public gene expression microarray data--methods and preliminary results. Radiology..

[CR40] Gillies RJ, Kinahan PE, Hricak H (2016). Radiomics: images are more than pictures, they are data. Radiology..

[CR41] Grootjans W, Tixier F, van der Vos CS, Vriens D, Le Rest CC, Bussink J et al (2016) The impact of optimal respiratory gating and image noise on evaluation of intratumor heterogeneity on ^18^F-FDG PET imaging of lung cancer. J Nucl Med 57(11):1692–1698 10.2967/jnumed.116.173112 27283931

[CR42] Grossmann P, Stringfield O, El-Hachem N, Bui MM, Rios Velazquez E, Parmar C (2017). Defining the biological basis of radiomic phenotypes in lung cancer. Elife..

[CR43] Hagendorff T (2020). The ethics of ai ethics: an evaluation of guidelines. Mind Mach.

[CR44] Halpenny DF, Riely GJ, Hayes S, Yu H, Zheng J, Moskowitz CS (2014). Are there imaging characteristics associated with lung adenocarcinomas harboring ALK rearrangements?. Lung Cancer.

[CR45] Handelman GS, Kok HK, Chandra RV, Razavi AH, Lee MJ, Asadi H (2018). eDoctor: machine learning and the future of medicine. J Intern Med.

[CR46] Hao H, Zhou Z, Li S, Maquilan G, Folkert MR, Iyengar P (2018). Shell feature: a new radiomics descriptor for predicting distant failure after radiotherapy in non-small cell lung cancer and cervix cancer. Phys Med Biol.

[CR47] Hawkins SH, Korecki JN, Balagurunathan Y, Gu Y, Kumar V, Basu S (2014). Predicting outcomes of nonsmall cell lung cancer using CT image features. IEEE Access.

[CR48] Holzinger A, Haibe-Kains B, Jurisica I (2019). Why imaging data alone is not enough: AI-based integration of imaging, omics, and clinical data. Eur J Nucl Med Mol Imaging.

[CR49] https://www.hunimed.eu/course/medtec-school/. Accessed on 26 Oct 2020

[CR50] https://www.radiologybusiness.com/topics/artificial-intelligence/wait-will-ai-replace-radiologists-after-all. Accessed on 26 Oct 2020

[CR51] Huang S, Yang J, Fong S, Zhao Q (2019). Mining prognosis index of brain metastases using artificial intelligence. Cancers (Basel).

[CR52] Hustinx R (2019). Physician centred imaging interpretation is dying out - why should I be a nuclear medicine physician?. Eur J Nucl Med Mol Imaging.

[CR53] Huynh E, Coroller TP, Narayan V, Agrawal V, Hou Y, Romano J (2016). CT-based radiomic analysis of stereotactic body radiation therapy patients with lung cancer. Radiother Oncol.

[CR54] Hyun SH, Ahn MS, Koh YW, Lee SJ (2019). A Machine-learning approach using PET-based radiomics to predict the histological subtypes of lung cancer. Clin Nucl Med.

[CR55] Ibrahim A, Primakov S, Beuque M, Woodruff HC, Halilaj I, Wu G (2020). Radiomics for precision medicine: Current challenges, future prospects, and the proposal of a new framework. Methods..

[CR56] Ibrahim A, Vallières M, Woodruff H, Primakov S, Beheshti M, Keek S, et al (2019) Radiomics Analysis for Clinical Decision Support in Nuclear Medicine. Seminars in Nuclear Medicine 49 10.1053/j.semnuclmed.2019.06.005 31470936

[CR57] James G, Witten D, Hastie T, Tibshirani R, James G, Witten D, Hastie T, Tibshirani R (2013). Statistical learning. An introduction to statistical learning: with applications in R.

[CR58] Jayasurya K, Fung G, Yu S, Dehing-Oberije C, De Ruysscher D, Hope A (2010). Comparison of Bayesian network and support vector machine models for two-year survival prediction in lung cancer patients treated with radiotherapy. Med Phys.

[CR59] Jemaa S, Fredrickson J, Carano RAD, Nielsen T, de Crespigny A, Bengtsson T (2020) Tumor segmentation and feature extraction from whole-body FDG-PET/CT using cascaded 2D and 3D convolutional neural networks. J Digit Imaging 10.1007/s10278-020-00341-1 PMC7522127 32378059

[CR60] Jiang M, Sun D, Guo Y, Guo Y, Xiao J, Wang L (2020). Assessing PD-L1 expression level by radiomic features from PET/CT in nonsmall cell lung cancer patients: an initial result. Acad Radiol.

[CR61] Jiang M, Zhang Y, Xu J, Ji M, Guo Y, Guo Y (2019). Assessing EGFR gene mutation status in non-small cell lung cancer with imaging features from PET/CT. Nucl Med Commun.

[CR62] Karami M (2015). Clinical decision support systems and medical imaging. Radiol Manage.

[CR63] Khorrami M, Khunger M, Zagouras A, Patil P, Thawani R, Bera K (2019). Combination of peri- and intratumoral radiomic features on baseline CT scans predicts response to chemotherapy in lung adenocarcinoma. Radiol Artif Intell.

[CR64] Kirienko M, Biroli M, Gelardi F, Seregni E, Chiti A, Sollini M (in Press) Where do we stand?

[CR65] Kirienko M, Cozzi L, Rossi A, Voulaz E, Antunovic L, Fogliata A (2018). Ability of FDG PET and CT radiomics features to differentiate between primary and metastatic lung lesions. Eur J Nucl Med Mol Imaging.

[CR66] Kirienko M, Sollini M, Silvestri G, Mognetti S, Voulaz E, Antunovic L (2018). Convolutional neural networks promising in lung cancer T-parameter assessment on baseline FDG-PET/CT. Contrast Media Mol Imaging.

[CR67] Kocak B, Kus EA, Kilickesmez O (2020) How to read and review papers on machine learning and artificial intelligence in radiology: a survival guide to key methodological concepts. Eur Radiol 10.1007/s00330-020-07324-4 33006018

[CR68] Koyasu S, Nishio M, Isoda H, Nakamoto Y, Togashi K (2019) Usefulness of gradient tree boosting for predicting histological subtype and EGFR mutation status of non-small cell lung cancer on ^18^F FDG-PET/CT. (2020) Ann Nucl Med 34:49–57. 10.1007/s12149-019-01414-0 31659591

[CR69] Lafata KJ, Hong JC, Geng R, Ackerson BG, Liu JG, Zhou Z (2019). Association of pre-treatment radiomic features with lung cancer recurrence following stereotactic body radiation therapy. Phys Med Biol.

[CR70] Lambin P, Leijenaar RTH, Deist TM, Peerlings J, de Jong EEC, van Timmeren J (2017). Radiomics: the bridge between medical imaging and personalized medicine. Nat Rev Clin Oncol.

[CR71] Leijenaar RT, Carvalho S, Velazquez ER, van Elmpt WJ, Parmar C, Hoekstra OS (2013). Stability of FDG-PET Radiomics features: an integrated analysis of test-retest and inter-observer variability. Acta Oncol.

[CR72] Leung KH, Marashdeh W, Wray R, Ashrafinia S, Pomper MG, Rahmim A et al (2020) A physics-guided modular deep-learning based automated framework for tumor segmentation in PET. Phys Med Biol 10.1088/1361-6560/ab8535 PMC12243949 32235059

[CR73] Li X, Yin G, Zhang Y, Dai D, Liu J, Chen P (2019). Predictive power of a radiomic signature based on ^18^F-FDG PET/CT images for EGFR mutational status in NSCLC. Front Oncol.

[CR74] Li XY, Xiong JF, Jia TY, Shen TL, Hou RP, Zhao J (2018). Detection of epithelial growth factor receptor. J Thorac Dis.

[CR75] Lian C, Ruan S, Denœux T, Jardin F, Vera P (2016). Selecting radiomic features from FDG-PET images for cancer treatment outcome prediction. Med Image Anal.

[CR76] Liao F, Liang M, Li Z, Hu X, Song S (2019). Evaluate the malignancy of pulmonary nodules using the 3-D deep leaky noisy-OR network. IEEE Trans Neural Netw Learn Syst.

[CR77] Luo Y, McShan DL, Matuszak MM, Ray D, Lawrence TS, Jolly S (2018). A multiobjective Bayesian networks approach for joint prediction of tumor local control and radiation pneumonitis in nonsmall-cell lung cancer (NSCLC) for response-adapted radiotherapy Jun 4:10.1002/mp.13029. Med Phys.

[CR78] Mandl KD, Glauser T, Krantz ID, Avillach P, Bartels A, Beggs AH (2020). The genomics research and innovation network: creating an interoperable, federated, genomics learning system. Genet Med.

[CR79] Minsky M, Papert S (1972). Perceptrons : an introduction to computational geometry.

[CR80] Montgomery DW, Amira A, Zaidi H (2007). Fully automated segmentation of oncological PET volumes using a combined multiscale and statistical model. Med Phys.

[CR81] Moore JH, Raghavachari N, Speakers W (2019). Artificial intelligence based approaches to identify molecular determinants of exceptional health and life span-an interdisciplinary workshop at the National Institute on Aging. Front Artif Intell.

[CR82] Nair VS, Gevaert O, Davidzon G, Napel S, Graves EE, Hoang CD et al (2012) Prognostic PET ^18^F-FDG uptake imaging features are associated with major oncogenomic alterations in patients with resected non-small cell lung cancer. Cancer Res 72(15):3725–3734 10.1158/0008-5472.CAN-11-3943 PMC3596510 22710433

[CR83] Nair VS, Gevaert O, Davidzon G, Plevritis SK, West R (2014) NF-κB protein expression associates with ^18^F-FDG PET tumor uptake in non-small cell lung cancer: a radiogenomics validation study to understand tumor metabolism. Lung Cancer 83(2):189–196 10.1016/j.lungcan.2013.11.001 PMC3922123 24355259

[CR84] Ninatti G, Kirienko M, Neri E, Sollini M, Chiti A (2020). Imaging-based prediction of molecular therapy targets in NSCLC by radiogenomics and AI approaches: a systematic review. Diagnostics (Basel).

[CR85] Ohri N, Duan F, Snyder BS, Wei B, Machtay M, Alavi A (2016). Pretreatment 18F-FDG PET textural features in locally advanced non-small cell lung cancer: secondary analysis of ACRIN 6668/RTOG 0235. J Nucl Med.

[CR86] Oikonomou A, Khalvati F, Tyrrell PN, Haider MA, Tarique U, Jimenez-Juan L (2018). Radiomics analysis at PET/CT contributes to prognosis of recurrence and survival in lung cancer treated with stereotactic body radiotherapy. Sci Rep.

[CR87] Oliver JA, Budzevich M, Zhang GG, Dilling TJ, Latifi K, Moros EG (2015). Variability of image features computed from conventional and respiratory-gated PET/CT images of lung cancer. Transl Oncol.

[CR88] Ongena YP, Haan M, Yakar D, Kwee TC (2020). Patients’ views on the implementation of artificial intelligence in radiology: development and validation of a standardized questionnaire. Eur Radiol.

[CR89] Parekh V, Jacobs MA (2016). Radiomics: a new application from established techniques. Expert Rev Precis Med Drug Dev.

[CR90] Paul R, Hawkins SH, Balagurunathan Y, Schabath MB, Gillies RJ, Hall LO (2016). Deep feature transfer learning in combination with traditional features predicts survival among patients with lung adenocarcinoma. Tomography..

[CR91] Perk T, Bradshaw T, Chen S, Im HJ, Cho S, Perlman S et al (2018) Automated classification of benign and malignant lesions in ^18^F-NaF PET/CT images using machine learning. Phys Med Biol 63(22):225019 10.1088/1361-6560/aaebd0 30457118

[CR92] Pesapane F, Volonté C, Codari M, Sardanelli F (2018). Artificial intelligence as a medical device in radiology: ethical and regulatory issues in Europe and the United States. Insights into Imaging.

[CR93] Pinto Dos Santos D, Giese D, Brodehl S, Chon SH, Staab W, Kleinert R (2019). Medical students' attitude towards artificial intelligence: a multicentre survey. Eur Radiol.

[CR94] Pons E, Braun LM, Hunink MG, Kors JA (2016). Natural language processing in radiology: a systematic review. Radiology..

[CR95] Poole D, Mackworth A, Goebel R (1998). Computational intelligence: a logical approach.

[CR96] Porenta G (2019). Is there value for artificial intelligence applications in molecular imaging and nuclear medicine?. J Nucl Med.

[CR97] Remedios S, Roy S, Blaber J, Bermudez C, Nath V, Patel MB (2019). Distributed deep learning for robust multi-site segmentation of CT imaging after traumatic brain injury. Proc SPIE Int Soc Opt Eng.

[CR98] Remedios SW, Roy S, Bermudez C, Patel MB, Butman JA, Landman BA (2020). Distributed deep learning across multisite datasets for generalized CT hemorrhage segmentation. Med Phys.

[CR99] Schmidhuber J (2015). Deep learning in neural networks: an overview. Neural Netw.

[CR100] Schwyzer M, Ferraro DA, Muehlematter UJ, Curioni-Fontecedro A, Huellner MW, von Schulthess GK (2018). Automated detection of lung cancer at ultralow dose PET/CT by deep neural networks - Initial results. Lung Cancer.

[CR101] Sheller MJ, Reina GA, Edwards B, Martin J, Bakas S (2019). Multi-institutional deep learning modeling without sharing patient data: a feasibility study on brain tumor segmentation. Brainlesion..

[CR102] Shen D, Wu G, Suk HI (2017). Deep Learning in Medical Image Analysis. Annu Rev Biomed Eng.

[CR103] Sibille L, Seifert R, Avramovic N, Vehren T, Spottiswoode B, Zuehlsdorff S (2020). F-FDG PET/CT uptake classification in lymphoma and lung cancer by using deep convolutional neural networks. Radiology..

[CR104] Sim I, Gorman P, Greenes RA, Haynes RB, Kaplan B, Lehmann H (2001). Clinical decision support systems for the practice of evidence-based medicine. J Am Med Inform Assoc.

[CR105] Smith JA, Abhari RE, Hussain Z, Heneghan C, Collins GS, Carr AJ (2020). Industry ties and evidence in public comments on the FDA framework for modifications to artificial intelligence/machine learning-based medical devices: a cross sectional study. BMJ Open.

[CR106] Sollini M, Antunovic L, Chiti A, Kirienko M (2019). Towards clinical application of image mining: a systematic review on artificial intelligence and radiomics. Eur J Nucl Med Mol Imaging.

[CR107] Sollini M, Bandera F, Kirienko M (2019). Quantitative imaging biomarkers in nuclear medicine: from SUV to image mining studies. Highlights from annals of nuclear medicine 2018. Eur J Nucl Med Mol Imaging.

[CR108] Sollini M, Cozzi L, Chiti A, Kirienko M (2018). Texture analysis and machine learning to characterize suspected thyroid nodules and differentiated thyroid cancer: where do we stand?. Eur J Radiol.

[CR109] Sollini M, Cozzi L, Ninatti G, Antunovic L, Cavinato L, Chiti A et al. (2020a) PET/CT radiomics in breast cancer: Mind the step. Methods S1046-2023(19)30263–4 10.1016/j.ymeth.2020.01.007 31978538

[CR110] Sollini M, Gelardi F, Matassa G, Delgado Bolton RC, Chiti A, Kirienko M (2020). Interdisciplinarity: an essential requirement for translation of radiomics research into clinical practice -a systematic review focused on thoracic oncology. Rev Esp Med Nucl Imagen Mol.

[CR111] Song SH, Park H, Lee G, Lee HY, Sohn I, Kim HS (2017). Imaging phenotyping using radiomics to predict micropapillary pattern within lung adenocarcinoma. J Thorac Oncol.

[CR112] Soufi M, Kamali-Asl A, Geramifar P, Rahmim A (2017) A novel framework for automated segmentation and labeling of homogeneous versus heterogeneous lung tumors in [ ^18^F]FDG-PET Imaging. Mol Imaging Biol 19(3):456–468 10.1007/s11307-016-1015-0 27770402

[CR113] Spyns P (1996). Natural language processing in medicine: an overview. Methods Inf Med.

[CR114] Stone M (1974). Cross-validatory choice and assessment of statistical predictions. J Royal Stat Soc Series B (Methodological).

[CR115] Suk H-I, Liu M, Yan P, Lian C. Machine Learning in Medical Imaging 10th International Workshop, MLMI 2019, Held in Conjunction with MICCAI 2019, Shenzhen, China, October 13, 2019, Proceedings: 10th International Workshop, MLMI 2019, Held in Conjunction with MICCAI 2019, Shenzhen, China, October 13, 2019, Proceedings 2019. 10.1007/978-3-030-35817-4_20 PMC7043018 32104792

[CR116] Sun YW, Xu J, Zhou J, Liu WJ (2018). Targeted drugs for systemic therapy of lung cancer with brain metastases. Oncotarget..

[CR117] Suzuki K (2012). A review of computer-aided diagnosis in thoracic and colonic imaging. Quant Imaging Med Surg.

[CR118] Tau N, Stundzia A, Yasufuku K, Hussey D, Metser U (2020). Convolutional neural networks in predicting nodal and distant metastatic potential of newly diagnosed non-small cell lung cancer on FDG PET images. AJR Am J Roentgenol.

[CR119] Thie JA (2004). Understanding the standardized uptake value, its methods, and implications for usage. J Nucl Med.

[CR120] Tixier F, Hatt M, Valla C, Fleury V, Lamour C, Ezzouhri S et al (2014) Visual versus quantitative assessment of intratumor ^18^F-FDG PET uptake heterogeneity: prognostic value in non-small cell lung cancer. J Nucl Med 55(8):1235–1241 10.2967/jnumed.113.133389 24904113

[CR121] Tseng H-H, Luo Y, Cui S, Chien J-T, Ten Haken RK, Naqa IE (2017). Deep reinforcement learning for automated radiation adaptation in lung cancer. Med Phys.

[CR122] Tzanoukos G, Athanasiadis E, Gaitanis A, Georgakopoulos A, Chatziioannou A, Chatziioannou S (2016). SPNsim: A database of simulated solitary pulmonary nodule PET/CT images facilitating computer aided diagnosis. J Biomed Inform.

[CR123] Tzanoukos G, Kafouris P, Georgakopoulos A, Gaitanis A, Maroulis D, Chatziioannou S (2019). Design and initial implementation of a computer aided diagnosis system for PET/CT solitary pulmonary nodule risk estimation. 2019 IEEE 19th International Conference on Bioinformatics and Bioengineering (BIBE).

[CR124] Vabalas A, Gowen E, Poliakoff E, Casson AJ (2019). Machine learning algorithm validation with a limited sample size. PLoS One.

[CR125] Vallières M, Freeman CR, Skamene SR, El Naqa I (2015). A radiomics model from joint FDG-PET and MRI texture features for the prediction of lung metastases in soft-tissue sarcomas of the extremities. Phys Med Biol.

[CR126] van Amsterdam WAC, Verhoeff JJC, de Jong PA, Leiner T, Eijkemans MJC (2019). Eliminating biasing signals in lung cancer images for prognosis predictions with deep learning. NPJ Digit Med.

[CR127] van Engelen JE, Hoos HH (2020). A survey on semi-supervised learning. Mach Learn.

[CR128] van Hoek J, Huber A, Leichtle A, Härmä K, Hilt D, von Tengg-Kobligk H (2019). A survey on the future of radiology among radiologists, medical students and surgeons: students and surgeons tend to be more skeptical about artificial intelligence and radiologists may fear that other disciplines take over. Eur J Radiol.

[CR129] van Velden FH, Kramer GM, Frings V, Nissen IA, Mulder ER, de Langen AJ et al (2016) Repeatability of radiomic features in non-small-cell lung cancer [^18^F]FDG-PET/CT studies: impact of reconstruction and delineation. Mol Imaging Biol 18(5):788–795 10.1007/s11307-016-0940-2 PMC5010602 26920355

[CR130] Varma S, Simon R (2006). Bias in error estimation when using cross-validation for model selection. BMC Bioinformatics.

[CR131] Vepakomma P, Gupta O, Swedish T, Raskar R (2018). Split learning for health: distributed deep learning without sharing raw patient data. arXiv.

[CR132] Volpp K, Mohta S (2016). Improved engagement leads to better ­outcomes, but better tools are needed.: NEJM Catalyst.

[CR133] Wang H, Zhou Z, Li Y, Chen Z, Lu P, Wang W (2017). Comparison of machine learning methods for classifying mediastinal lymph node metastasis of non-small cell lung cancer from. EJNMMI Res.

[CR134] Wang S, Shi J, Ye Z, Dong D, Yu D, Zhou M (2019). Predicting EGFR mutation status in lung adenocarcinoma on computed tomography image using deep learning. Eur Respir J.

[CR135] Wang S, Zhou M, Liu Z, Gu D, Zang Y, Dong D (2017). Central focused convolutional neural networks: Developing a data-driven model for lung nodule segmentation. Med Image Anal.

[CR136] Wang X, Kong C, Xu W, Yang S, Shi D, Zhang J (2019). Decoding tumor mutation burden and driver mutations in early stage lung adenocarcinoma using CT-based radiomics signature. Thorac Cancer.

[CR137] Way GP, Allaway RJ, Bouley SJ, Fadul CE, Sanchez Y, Greene CS (2017). A machine learning classifier trained on cancer transcriptomes detects NF1 inactivation signal in glioblastoma. BMC Genomics.

[CR138] Weikert T, Akinci D'Antonoli T, Bremerich J, Stieltjes B, Sommer G, Sauter AW (2019). Evaluation of an AI-powered lung nodule algorithm for detection and 3D segmentation of primary lung tumors. Contrast Media Mol Imaging.

[CR139] Weiss GJ, Ganeshan B, Miles KA, Campbell DH, Cheung PY, Frank S (2014). Noninvasive image texture analysis differentiates K-ras mutation from pan-wildtype NSCLC and is prognostic. PLoS One.

[CR140] Wilkinson MD, Dumontier M, Aalbersberg IJ, Appleton G, Axton M, Baak A (2016). The FAIR guiding principles for scientific data management and stewardship. Sci Data.

[CR141] Wu W, Parmar C, Grossmann P, Quackenbush J, Lambin P, Bussink J (2016). Exploratory study to identify radiomics classifiers for lung cancer histology. Front Oncol.

[CR142] Wu Y, Liu J, Han C, Liu X, Chong Y, Wang Z (2020). Preoperative prediction of lymph node metastasis in patients with early-T-stage non-small cell lung cancer by machine learning algorithms. Front Oncol.

[CR143] Xiong JF, Jia TY, Li XY, Yu W, Xu ZY, Cai XW (2018). Identifying epidermal growth factor receptor mutation status in patients with lung adenocarcinoma by three-dimensional convolutional neural networks. Br J Radiol.

[CR144] Xu Y, Hosny A, Zeleznik R, Parmar C, Coroller T, Franco I (2019). Deep learning predicts lung cancer treatment response from serial medical imaging. Clin Cancer Res.

[CR145] Yoon HJ, Sohn I, Cho JH, Lee HY, Kim JH, Choi YL (2015). Decoding tumor phenotypes for ALK, ROS1, and RET fusions in lung adenocarcinoma using a radiomics approach. Medicine (Baltimore).

[CR146] Yoon J, Suh YJ, Han K, Cho H, Lee HJ, Hur J (2020). Utility of CT radiomics for prediction of PD-L1 expression in advanced lung adenocarcinomas. Thorac Cancer.

[CR147] Ypsilantis PP, Siddique M, Sohn HM, Davies A, Cook G, Goh V (2015). Predicting response to neoadjuvant chemotherapy with PET imaging using convolutional neural networks. PLoS One.

[CR148] Zhang H, Molitoris J, Tan S, Giacomelli I, Scartoni D, Gzell C (2016). SU-F-R-04: radiomics for survival prediction in glioblastoma (GBM). Med Phys.

[CR149] Zhang L, Chen B, Liu X, Song J, Fang M, Hu C (2018). Quantitative biomarkers for prediction of epidermal growth factor receptor mutation in non-small cell lung cancer. Transl Oncol.

[CR150] Zhang R, Cheng C, Zhao X, Li X (2019). Multiscale mask R-CNN-based lung tumor detection using PET imaging. Mol Imaging.

[CR151] Zhao W, Yang J, Ni B, Bi D, Sun Y, Xu M (2019). Toward automatic prediction of EGFR mutation status in pulmonary adenocarcinoma with 3D deep learning. Cancer Med.

[CR152] Zittrain J. Ethics and governance of artificial intelligence. https://www.media.mit.edu/groups/ethics-and-governance/overview/. Accessed on 26 Oct 2020

